# Supplementary material for: Content-rich biological network constructed by mining PubMed abstracts
Source: BMC Bioinformatics. 2004 Oct 8;5:147. doi: 10.1186/1471-2105-5-147 (PMC528731; doi:10.1186/1471-2105-5-147)
Supplement: Additional File 2 — The original results of the above study (non-essential files are deleted to keep the file size under the limit set by BMC bioinformatics). [file 1471-2105-5-147-S2.bz2 › chilibotAdditionalFile2/dip05/362ID8558064E5750/html/IL8_FY.html]

 


 **IL8** and **FY** 
  
Found 25 abstracts in PubMed, retrieved 05.  
 

 What does Google say? 
 PDF only 
| .edu only 

---

**Interactive relationship** (e.g. stimulation, inhibition, etc)

**Inhibitory relationship**- Binding of IL 8  [ **IL8** ]  to both rheumatoid and non rheumatoid synovia was significantly reduced in the presence of anti DARC  [ **FY** ]  Fy3 and Fy6 monoclonal antibodies.  Ref: 12081195 J Pathol, 2002
**Neutral relationship**- The red blood cell Duffy antigen receptor for chemokines  [ **FY** ]  serves as a sink for the clearance of chemokines such as interleukin 8  [ **IL8** ]  IL 8  [ **IL8** ]  from the circulation.  Ref: 10964545 Clin Immunol, 2000

**Non-interactive relationship** (e.g. studied together, co-existance, homology, etc.)

- We also determined serum tumor necrosis factor alpha TNF alpha, the phenotypes of the IL 8  [ **IL8** ]  binding Duffy blood group  [ **FY** ]  antigen receptor and the von Willebrand factor vWF plasma levels.  Ref: 12516654 Am J Reprod Immunol, 2002
- The involvement of DARC  [ **FY** ]  in chemokine binding was studied by incubating sections with blocking antibodies to DARC  [ **FY** ]  Fy3 and 6, to find out if these reduced 125I IL 8  [ **IL8** ]  binding.  Ref: 12081195 J Pathol, 2002
- The serum levels of IL 8  [ **IL8** ]  and monocyte chemotactic peptide MCP 1, two DARC  [ **FY** ]  binding chemokines, were investigated and related to this polymorphism.  Ref: 12739041 Rheumatol Int, 2003
